# Abuse of Alcohol

**Published:** 1865-12

**Authors:** 


### ABUSE OF ALCOHOL.

---

In a paper communicated to the French Academy of Medicine by M. Lancereaux, on the changes produced by the abuse of alcohol, he observes that, since 1858, he had been engaged in tracing out the specific anatomical characters due to the action of certain agents. Last year, he did this with regard to the poison of syphilis, and now he gives the result of his investigations on alcohol. Accidental or acute alcoholism is found connected with special forms of ordinary affections. Thus, Tardieu has referred to its agency certain cases of hemorrhage of the membranes and ventricles of the brain and of the lungs; and some of the inflamations with rapid suppuration of the lungs and liver, and more rarely of the brain, derive their rapidly fatal character from the absorption of too much alcohol. But it is to lesions connected with prolonged abuse of this fluid that M. Lancereaux especially directs attention, and these he thinks may be conveniently considered under two principal heads. In one of these the organic connective tissue is principally concerned, giving rise to affections analogous to the adhesive inflammation of Hunter; and in the oth r, special modifications of the functionary element are produced, giving rise to lesions generally known as fatty degeneration.

The first of these alterations spares but few organs, invading the recesses of the parenchyma, or developing itself on the surface of the membranes; but the liver, brain, kidneys, and serous membranes, are the portions of the frame especially attacked. The liver may be taken as the model organ for exhibiting its effects, and the changes operated in it have been characterized by the English writers under the term cirrhosis. In thirty-five cases, M. Lancereaux has always observed precisely the same characters, differing only in their degree of evolution. A very analogous alteration is observed in the brain, this organ diminishing gradually in size, the convolutions undergoing atrophy, especially at the upper portion of the hemisphere, the brain also becoming colorless, and acquiring a firmer consistence. The arachnoid and pia mater are usually at the same time infiltrated, thickened, and strewed with whitish points or plates. The kidneys sometimes exhibit a condition analogous to that of the liver; but M. Lancereaux has not observed the condition of chronic pneumonia described by Huss. The mucous membranes of the stomach and cæcum are especially characterized by injection and hemorrhagic punctuation, appearances also observed in the bronchial and especially the laryngeal mucous membrane. Finally the coats of the vessels which especially come in contact with the products of absorption exhibit deposits capable of contracting or even obliterating their cavities. This is the case with the vena portæ and the pulmonary artery.

The alterations of the second class are characterized by the presence of fatty degeneration; and here, too, the liver is especially the organ affected, assuming an augmented size, but in its antero-posterior direction, which gives it a cubic form distinctive from other forms of fatty degeneration. The kidneys, pancreas, the salivary and gastric glands, the bronchial epithelium, and even the spermatic canals, may all exhibit this degeneration; the muscular fibre, bones, and cartilages, participating in the change. The relative frequency of the two orders of alterations is not constant; for while fatty degeneration may be said to be constant in certain organs, as the liver, chronic adhesive inflamation has only been met with thirty-five times in 130 cases; but one point is observable, that while adhesive inflamations have for the most part been observed in persons pursuing laborious callings, fatty degeneration has almost always been met with in the sedentary. With respect to the mode of production of these different lesions, it may be suggested that the adhesive inflamations are due to the more direct action of the alcohol on the tissues, the effect of which is not very different from that produced by the injection of alcoholic fluids into the tunica vaginalis. The chief seats of the lesion would seem to support this view, since the organs which are most directly influenced, as the vena portæ, liver and pulmonary artery, are also those which are oftenest attacked. The fatty degeneration would seem to be dependent on the impaired nutrition, itself connected with the diminution of the exhalation of carbonic acid.

"An important point to be noticed is the resemblance which this latter alteration bears to that consequent on the progress of age. In the drunkard, as well as in the aged, we have progress atrophy of the brain, increase of the cerebrospinal fluid, fatty degeneration of the minute vessels, of the substance of the heart, and of most of the anatomical elements, dilation of the pulmonary vesicles, ossification of the costal cartilages, and a substitution of fatty for osseous substance. So close is this resemblance, that it may be stated without exaggeration that, in the majority of cases, alcoholism produces an anticipated senility. What is true in physiology holds good also in pathology; for in the course of most diseases, and especially of acute diseases, there are exhibited in the drunkard and the aged person but few differences in the mode of action of the nervous system and in the power of the economy.—*Medical Circular.*

